# Cost-effectiveness of personalized medical treatment in acromegaly: a post hoc analysis of the ACROFAST study

**DOI:** 10.1210/jendso/bvag030

**Published:** 2026-03-06

**Authors:** Montserrat Marques-Pamies, Laura Ricou, Joan Gil, Miguel Sampedro-Núñez, Betina Biagetti, Olga Giménez-Palop, Marta Hernández, Silvia Martínez, Cristina Carrato, Rocío Villar-Taibo, Marta Araujo-Castro, Concepción Blanco, Inmaculada Simón-Muela, Andreu Simó-Servat, Gemma Xifra, Federico Vázquez, Isabel Pavón, José Antonio Rosado, Rogelio García-Centeno, Roxana Zavala, Felicia Alexandra Hanzu, Mireia Mora, Anna Aulinas, Nuria Vilarrasa, Alberto Torres, Soledad Librizzi, María Calatayud, Paz de Miguel Novoa, Cristina Alvarez-Escola, Antonio Picó, Isabel Salinas, Carmen Fajardo-Montañana, Rosa Cámara, Ignacio Bernabéu, Mireia Jordà, Susan M Webb, Mónica Marazuela, Elena Valassi, Manel Puig-Domingo

**Affiliations:** Department of Endocrinology and Nutrition, Hospital General de Granollers, 08402 Granollers, Spain; Research Group on Innovation, Health Economics and Digital Transformation—Institut Germans Trias I Pujol, CRES—Pompeu Fabra University, 08916 Barcelona, Spain; Endocrine Research Unit, Germans Trias I Pujol Research Institute (IGTP), 08916 Badalona, Spain; Centro de Investigación Biomédica en Red de Enfermedades Raras (CIBERER, Unidad 747), Instituto de Salud Carlos III (ISCIII), 28029 Madrid, Spain; Department of Endocrinology and Nutrition, EndoERN, Research Center for Pituitary Diseases, Institut de Recerca Sant Pau (IIB-Sant Pau), Hospital Sant Pau, 08025 Barcelona, Spain; Department of Endocrinology and Nutrition, La Princesa University Hospital, 28006 Madrid, Spain; Endocrine Research Unit, Instituto de Investigación del Hospital de La Princesa, 28006 Madrid, Spain; Centro de Investigación Biomédica en Red de Enfermedades Raras (CIBERER, Unidad 747), Instituto de Salud Carlos III (ISCIII), 28029 Madrid, Spain; Department of Endocrinology and Nutrition, Vall Hebron University Hospital, 08035 Barcelona, Spain; Department of Endocrinology and Nutrition, Hospital Dos de Maig, 08025 Barcelona, Spain; Department of Endocrinology and Nutrition, Arnau de Vilanova University Hospital, 25198 Lleida, Spain; Endocrine Research Unit, Lleida Institute for Biomedical Research Dr. Pifarré Foundation (IRBLleida), 25198 Lleida, Spain; Department Hormonal Laboratory, Germans Trias I Pujol University Hospital, 08916 Badalona, Spain; Department of Pathology, Germans Trias I Pujol University Hospital, 08916 Badalona, Spain; Department of Endocrinology and Nutrition, Clínico de Santiago University Hospital, 15706 Santiago de Compostela, Spain; Department of Endocrinology and Nutrition, Ramón y Cajal University Hospital, 28034 Madrid, Spain; Endocrine Research Unit, Instituto de Investigación Ramón y Cajal (IRYCIS), 28034 Madrid, Spain; Department of Endocrinology and Nutrition, Príncipe de Asturias University Hospital, 28805 Alcalà de Henares, Spain; Department of Endocrinology and Nutrition, Joan XXIII University Hospital, 43005 Tarragona, Spain; Endocrine Research Unit, Institut d´Investigació Sanitària Pere Virgili (IISPV), 43005 Tarragona, Spain; Departament of Medicine, Rovira I Virgili University (URV), 43003 Tarragona, Spain; Endocrine Research Unit, Institut D’Investigació Biomèdica de Bellvitge (IDIBELL), 08908 Hospitalet de Llobregat, Spain; Department of Endocrinology and Nutrition, Mutua de Terrassa University Hospital, 08221 Terrassa, Spain; Department of Endocrinology and Nutrition, Josep Trueta University Hospital, 17007 Girona, Spain; Department of Endocrinology and Nutrition, Germans Trias I Pujol University Hospital, 08916 Badalona, Spain; Department of Endocrinology and Nutrition, Getafe University Hospital, 28905 Getafe, Spain; Department of Endocrinology and Nutrition, Getafe University Hospital, 28905 Getafe, Spain; Department of Endocrinology and Nutrition, Gregorio Marañón University Hospital, 28007 Madrid, Spain; Department of Endocrinology and Nutrition, Joan XXIII University Hospital, 43005 Tarragona, Spain; Department of Endocrinology and Nutrition, Hospital Clinic University Hospital, 08036 Barcelona, Spain; Endocrine Research Unit, Institut D’Investigacions Biomèdiques August Pi I Sunyer (IDIBAPS), 08036 Barcelona, Spain; Departament of Medicine, Universitat de Barcelona (UB), 08036 Barcelona, Spain; Department of Endocrinology and Nutrition, Hospital Clinic University Hospital, 08036 Barcelona, Spain; Endocrine Research Unit, Institut D’Investigacions Biomèdiques August Pi I Sunyer (IDIBAPS), 08036 Barcelona, Spain; Departament of Medicine, Universitat de Barcelona (UB), 08036 Barcelona, Spain; Centro de Investigación Biomédica en Red de Enfermedades Raras (CIBERER, Unidad 747), Instituto de Salud Carlos III (ISCIII), 28029 Madrid, Spain; Department of Endocrinology and Nutrition, EndoERN, Research Center for Pituitary Diseases, Institut de Recerca Sant Pau (IIB-Sant Pau), Hospital Sant Pau, 08025 Barcelona, Spain; Endocrine Research Unit, Institut D’Investigació Biomèdica de Bellvitge (IDIBELL), 08908 Hospitalet de Llobregat, Spain; Department of Endocrinology and Nutrition, Bellvitge University Hospital, 08907 Hospitalet de Llobregat, Spain; Centro de Investigación Biomédica en Red de Diabetes y Enfermedades Metabólicas (CIBERDEM), Instituto de Salud Carlos III (ISCIII), 28029 Madrid, Spain; Department of Neurosurgery, Bellvitge University Hospital, 08907 Bellvitge, Spain; Department of Endocrinology and Nutrition, 12 de Octubre University Hospital, 28041 Madrid, Spain; Department of Endocrinology and Nutrition, 12 de Octubre University Hospital, 28041 Madrid, Spain; Department of Endocrinology and Nutrition, Clínico San Carlos University Hospital, 28040 Madrid, Spain; Department of Endocrinology and Nutrition, La Paz University Hospital, 28045 Madrid, Spain; Department of Endocrinology and Nutrition, General University Hospital Dr Balmis, Miguel Hernández University, 03010 Alicante, Spain; Endocrine Research Unit, Instituto de Investigación Sanitaria y Biomédica de Alicante (ISABIAL), 03010 Alicante, Spain; Department of Endocrinology and Nutrition, Getafe University Hospital, 28905 Getafe, Spain; Department of Endocrinology and Nutrition, La Ribera University Hospital, 46600 Valencia, Spain; Department of Endocrinology and Nutrition, La Fe University Hospital, 46026 Valencia, Spain; Department of Endocrinology and Nutrition, Ramón y Cajal University Hospital, 28034 Madrid, Spain; Endocrine Research Unit, Germans Trias I Pujol Research Institute (IGTP), 08916 Badalona, Spain; Centro de Investigación Biomédica en Red de Enfermedades Raras (CIBERER, Unidad 747), Instituto de Salud Carlos III (ISCIII), 28029 Madrid, Spain; Department of Endocrinology and Nutrition, EndoERN, Research Center for Pituitary Diseases, Institut de Recerca Sant Pau (IIB-Sant Pau), Hospital Sant Pau, 08025 Barcelona, Spain; Departament of Medicine, Universitat Autònoma de Barcelona (UAB), 08193 Bellaterra, Spain; Department of Endocrinology and Nutrition, La Princesa University Hospital, 28006 Madrid, Spain; Endocrine Research Unit, Instituto de Investigación del Hospital de La Princesa, 28006 Madrid, Spain; Endocrine Research Unit, Germans Trias I Pujol Research Institute (IGTP), 08916 Badalona, Spain; Centro de Investigación Biomédica en Red de Enfermedades Raras (CIBERER, Unidad 747), Instituto de Salud Carlos III (ISCIII), 28029 Madrid, Spain; Department of Endocrinology and Nutrition, Getafe University Hospital, 28905 Getafe, Spain; Endocrine Research Unit, Germans Trias I Pujol Research Institute (IGTP), 08916 Badalona, Spain; Centro de Investigación Biomédica en Red de Enfermedades Raras (CIBERER, Unidad 747), Instituto de Salud Carlos III (ISCIII), 28029 Madrid, Spain; Department of Endocrinology and Nutrition, Getafe University Hospital, 28905 Getafe, Spain; Departament of Medicine, Universitat Autònoma de Barcelona (UAB), 08193 Bellaterra, Spain

**Keywords:** acromegaly, medical treatment, personalized therapy, first-generation somatostatin receptor ligands, therapeutic response prediction, clinical trial, cost-effectiveness

## Abstract

**Context:**

Medical treatment of acromegaly is currently performed through a trial-and-error approach using first-generation somatostatin receptor ligands (fgSRLs) as first-line drugs, with an efficacy of 50%. Some biomarkers can predict patient response, potentially benefiting nonresponders by using personalized treatment approaches. The ACROFAST study revealed that the probability of disease control was 2.53 times higher (CI 1.30-4.80) with personalized treatment, and insulin-like growth factor 1 (IGF1) normalization occurred in 78% vs 53% of patients, compared with standard treatment (*P* < .05).

**Objective:**

To evaluate the cost-effectiveness of personalized medicine based on predictive biomarkers, from the results of the ACROFAST study.

**Methods:**

Post hoc analysis from the ACROFAST study comparing cost-effectiveness of a protocol of medical treatment based on predictive biomarkers to fgSRLs (n = 32) vs standard treatment (n = 36) for 12 months. Costs analyzed were visits, examinations, study of biomarkers (121€/patient), and pharmacological treatment according to the prices established by the Spanish National Health Care System. Medium-term costs were evaluated, considering a projection of up to 2 years in uncontrolled cases, assuming positive and negative scenarios with additional monitoring costs and a 25% likelihood of disease progression per follow-up visit.

**Results:**

The personalized protocol reduced the average cost per patient required to achieve disease control by 22% (15 127€ vs 19 420€). These results represent an annual saving of 15 263€ per patient achieving hormonal control compared to the standard treatment.

**Conclusion:**

Personalized medicine, using a relatively straightforward biomarker-based protocol, enables a greater proportion of patients to attain hormonal control and proves to be a cost-effective strategy for managing acromegaly.

Acromegaly is a rare, chronic disorder characterized by excessive secretion of growth hormone (GH) and consequent elevation of insulin-like growth factor I (IGF1) levels, typically resulting from a pituitary adenoma [1-3]. Standard treatment modalities, including surgery, first-generation somatostatin receptor ligands (fgSRLs), GH receptor antagonists, and radiotherapy, aim to control hormone hypersecretion, alleviate symptoms, and prevent long-term complications [4, 5]. However, the heterogeneous nature of the disease and patients' variability in response to treatment highlight the limitations of a uniform therapeutic approach [6-9]. Personalized treatment protocols tailored to individual patient profiles have emerged as a promising alternative, aiming to optimize clinical outcomes while balancing economic considerations [6, 7, 10, 11].

The recently published ACROFAST study [12] introduced a novel personalized protocol for treating acromegaly, integrating patient-specific factors, such as tumor characteristics, biochemical response patterns, and comorbidities, to guide therapeutic decisions. Findings from ACROFAST indicated that this personalized approach may offer superior effectiveness by achieving IGF1 normalization in a shorter period of time. Moreover, the individualized approach could potentially reduce overtreatment, minimize adverse effects, and maximize patient satisfaction.

The present post hoc study of ACROFAST aims to evaluate the superiority of the personalized treatment protocol over standard treatment for acromegaly by examining cost-effectiveness. Medical standard treatment protocols based on the first-line use of fgSRLs have historically provided insufficient biochemical control rates [13-15], inconsistent symptom management, and varying impacts on patients' daily lives. Therefore, they may consequently be associated with higher healthcare costs. In contrast, personalized protocols try to address these shortcomings by adjusting treatment intensity based on individual needs. This may enhance therapeutic efficiency as well as decrease the general costs of acromegaly treatment.

In this context, the ACROFAST study serves as a critical benchmark to change the paradigm in acromegaly management, offering insights into the clinical and economic benefits of personalization. This research aims to elucidate the potential advantages of personalized protocols in terms of cost-effectiveness, paving the way for its broader implementation in clinical practice with the ultimate aim to improve medical management of patients living with acromegaly.

## Methods

### Study design

The design of the ACROFAST study and their main results have been described previously [12]. In brief, this was a prospective, randomized clinical trial comparing a protocol based on predictive biomarkers of fgSRLs vs standard treatment, in 2 parallel arms in which 21 Spanish university hospitals participated comparing the effectiveness and time to control of 2 treatment protocols for 12 months. A total of 85 patients participated and 68 patients completed the study and were evaluated: 32 in the personalized arm and 36 in the control group, either before or after unsuccessful surgery. In the personalized protocol, patients were treated with fgSRLs as monotherapy, fgSRLs in combination with pegvisomant, or pegvisomant as monotherapy, depending on the results obtained in the short Acute Octreotide Test (sAOT), tumor T2 magnetic resonance imaging (MRI) signal, or immunostaining for E-cadherin (mouse monoclonal anti-E-cadherin antibody [RRID AB_397580] [Ventana, Tucson, Ariz., USA] purchased as a prediluted antibody, with a concentration of 0.314 μg/dL). Serum GH was measured at each center by different automated immunoassays, all calibrated against World Health Organization (WHO) International Standard 98/574: Immulite i2000, Siemens Healthineers (RRID:AB_2811291); Liason XL, Diasorin (RRID:AB_3099571); UniCel DxI 800 Access, Beckman Coulter (RRID:AB_2756876) and Cobas 8000, Roche Diagnostics (RRID:AB_2883974). To ensure consistency and comparability of GH measurements obtained from different immunoassays and centers, the results were harmonized as previously described [12]. Serum IGF1 concentrations were also measured in each center by immunoassays calibrated against WHO NISBC 2stIS 02/254: Liason XL, Diasorin (RRID: AB_2928957), Immulite i2000, Siemens Healthineers (RRID: AB_2922766) and ELISA Mediagnost (RRID: AB_2813791). IGF1 concentrations were evaluated as absolute concentrations, and they were calculated as IGF1 SD score (SDS) for outcomes assessment and inter-center comparability as previously described [12]. In the control group the medical treatment always started with fgSRLs, and the other drugs were included after demonstrating inadequate control with those previously used, according to clinical judgment based on the standard of care. The main variables included were the time to control in each group, analyzed with a Survival Analysis, and the proportion of controlled patients at each visit and at the end of the study.

The primary measure of the present study was the monthly and the total cost of therapy for each treatment arm (12 months of study period). The required doses of fgSRLs and pegvisomant for all participants were assessed, and the monthly treatment costs were calculated based on the average wholesale price (AWP) in Spain for each dosing regimen (Table 1). Since the fgSRL dose followed a specific escalation per protocol within each treatment arm, the dose was multiplied by its respective AWP. For pegvisomant, the mean ± SD dose required by all patients in each of the treatment arms was determined.

**Table 1:** Costs associated with both treatment protocols at different follow-up visits

	Standard treatment	Personalized treatment	*P* value
	Cost per patient		
Biomarkers study			
Acute octreotide test	—	8€	
E-cadherin immunostaining	—	15€	
Medical visit	—	98€	
First follow-up			
Medication	2726€	4359€	
Medical visit	98€	98€	
Blood analyses (hematology, biochemistry, and hormones)	18€	18€	
MRI	226€	226€	
Second follow-up			
Medication	3544€	7107€	
Medical visit	98€	98€	
Blood analyses (hematology, biochemistry, and hormones)	18€	18€	
MRI	226€	226€	
Third follow-up			
Medication	9782€	9191€	
Medical visit	98€	98€	
Blood analyses (hematology, biochemistry, and hormones)	18€	18€	
MRI	226€	226€	
Fourth follow-up			
Medication	15 846€	9191€	
Medical visit	98€	98€	
Blood analyses (hematology, biochemistry, and hormones)	18€	18€	
MRI	226€	226€	
Percentage of patients achieving hormonal control			
3 months follow-up	25%	38%	.43
6 months follow-up	47%	69%	.14
9 months follow-up	50%	72%	.13
12 months follow-up	50%	78%	.04

**Figure 1:**
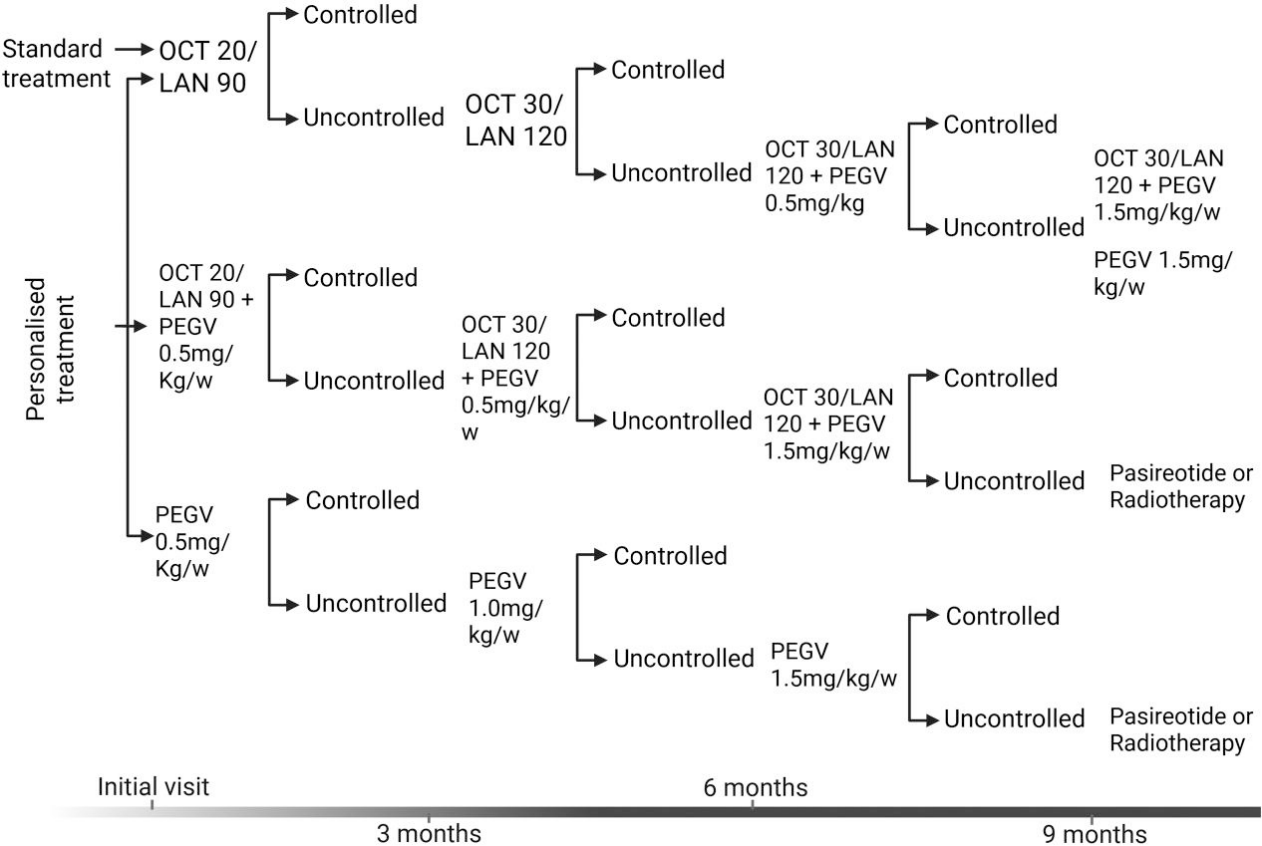
Algorithm for treatment escalation in standard and personalized arms. OCT (octreotide), LAN (lanreotide), PEGV (pegvisomant). Doses of octreotide: 20 and 30 mg administered every 4 weeks. Doses of lanreotide: 90 and 120 mg administered every 4 weeks. Doses of pegvisomant were calculated according to the weight of each patient at a weekly dose of 0.5 mg, 1.0 and 1.5 mg/Kg/w.

The study utilized a cost-effectiveness analysis (CEA) to compare a personalized treatment protocol vs standard medical care in patients with acromegaly. The comparison was made using a decision tree model from the perspective of the healthcare provider, considering a 1-year time horizon. The model specified dose escalation for each therapeutic modality as shown in Fig. 1.

The costs associated with each treatment alternative were estimated according to the costs in Spain in 2024. In terms of effectiveness, the proportion of controlled and uncontrolled patients at the end of the study period was compared. The control of the disease was defined when gender and age adjusted IGF1 levels achieved < 2.5 SDS [16]. The results were expressed in terms of incremental cost-effectiveness ratio (ICER). The analysis followed the standards established in the CHEERS (Consolidated Health Economic Evaluation Reporting Standards) guide [17].

In addition, the medium-term results were estimated, considering a time horizon of 12 months as minimum and 24 months as maximum from the beginning of the treatment; as the uncontrolled patients, mostly from the standard algorithm, required an escalation of treatment which was performed following standard clinical practice. This analysis included the calculation of the additional costs associated with an uncontrolled patient case per year, as well as a projection of the evolution of the proportion of patients controlled in this period following the behavioral model obtained in the study period of the first year of the ACROFAST data. To make this estimate, 2 scenarios were defined for each type of treatment:

- Positive scenario: Assuming a 25% increase in the proportion of patients controlled with respect to the average observed in the first year of the study in each follow-up visit performed at 3 months interval.
- Negative scenario: Assuming a 25% decrease in the proportion of patients controlled with respect to the average observed in the first year of the study.

These projections allowed the sensitivity of the economic and effectiveness results to be evaluated in the face of possible variations in the evolution of patient control.

### Cost data

The direct costs of both treatments were estimated, including drugs, scheduled and nonscheduled visits, tests, imaging procedures, and biochemical analyses carried out during the study period. The cost data was obtained from the institution's administrative databases and was measured in monetary units (€2024). A discount rate was not applied due to the 1-year time limit of the study.

The effectiveness values, represented by the proportion of patients achieving hormonal control monitored in each phase of treatment, were obtained from the results of the patients in the study sample.

### Statistical analysis

The ACROFAST trial was designed with 80% power to detect a difference of 1 SD in the effectiveness between any 2 study arms (effect size = 1.0), using an independent samples *t* test with a 2-sided alpha level of 5%. Forty participants per arm were required to meet these parameters. Numerical variables were summarized using mean, median, SD, and range. Categorical variables were summarized as frequencies and percentages and compared across treatment arms using Fisher's exact test.

Costs across treatment arms were analyzed using analysis of variance (ANOVA) with Tukey post hoc tests and simultaneous 95% CIs. Mean total monthly costs were calculated and compared accordingly. The Tukey test was used to perform pairwise comparisons between group means while controlling for the risk of type I error (false positives) in the context of multiple comparisons. In the cost-effectiveness analysis, this test was applied for analyzing the costs across multiple treatment groups to identify which specific pairs of groups had statistically significant differences in their mean costs. A 2-sided significance level of 0.05 was applied throughout the analysis. Statistical computations were performed using the statistical program R version 4.4.0 (2024-06-14) for Windows, while figures were generated using BioRender.com.

## Results

**Figure 2:**
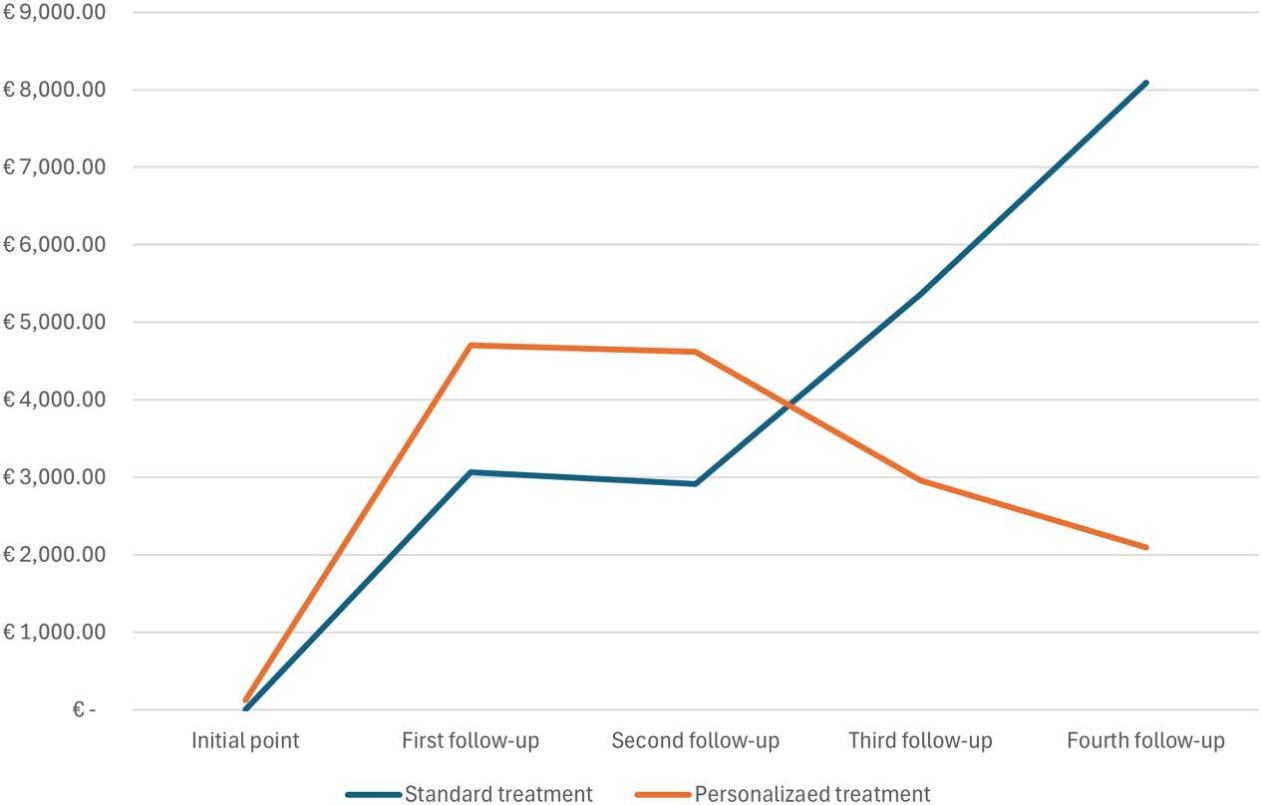
Cumulative line graph of costs in standard and personalized protocol treatment.

The ACROFAST study included a total of 85 patients. From these, 68 patients ended the study and were finally analyzed: 32 patients in the personalized protocol and 36 patients in the standard protocol. Results of the ACROFAST study showed that the probability of achieving hormonal control adjusted by age and sex in the personalized group was defined by a hazard ratio of 2.53 (CI 1.30-4.80). Survival analysis with the cumulative number of controlled patients is available in the results of the ACROFAST study [12]. At the end of the study period, personalized treatment achieved 25% of more controlled patients compared to standard treatment (78% vs 53%), which implied a 22% reduction in the average cost per patient with a personalized treatment compared to standard treatment (15 127€ vs 19 420€) (Table 1). Cumulative line graph of costs in controlled and uncontrolled patients is represented in Fig. 2.

Personalized treatment generated an initial additional cost due to the sAOT, the E-cadherin immunostaining in postsurgical patients, and the medical visit associated with the result evaluation for assigning the specific medical treatment, totaling a cost of 121€ per patient. The cost of the sAOT was 8€. It included the cost of the medication, the GH evaluation at baseline and at 2 hours, and the cost of the professional who performed the 2 blood extractions. We did not include the cost of the administration process, as it is not established within the healthcare system and it was not necessary to hire additional resources beyond those already available. It also implied a higher pharmacological cost in the first 2 controls (12 150€ vs 6955€) since a proportion of patients were treated with pegvisomant in monotherapy or in combination with fgSRLs in the personalized arm. However, these costs decreased in the final stages of treatment (19 067€ vs 26 311€, in the last 2 follow-up visits). In contrast, the costs of medication in the conventional treatment arm increased progressively over time, as more therapeutic changes were required in the uncontrolled patients. Furthermore, the personalized treatment achieved a higher percentage of patients with hormonal control from the first follow-up evaluation (38% vs 25%) which increased steadily compared to the standard treatment arm at the last control visit (78% vs 53%). This higher level of effectiveness from the early stages reduced the number of patients who progress to more expensive stages, thus contributing to a lower total cost per patient at the end of the study (Table 2).

**Table 2:** Estimation of average differential cost/year between personalized and standard treatment for acromegaly in a theoretical given patient of each group

	Standard treatment	Personalized treatment	Δ
Average cost per patient	19 420€	15 127€	−4293€
Percentage of patients achieving hormonal control	50%	78%	28%
ICER	−15 263€ Per additional patient achieving hormonal control		

Abbreviation: ICER, incremental cost-effectiveness ratio

Thus, the personalized treatment reduced costs by 4293€ per patient/year and increased the effectiveness of the treatment, with 25% of more patients achieving hormonal control in less time in the personalized arm. These results represented a reduction of 15 263€ per additional patient monitored with the personalized treatment compared to the standard treatment.

When a 24-month projection was performed, the results showed that, in the medium term, personalized treatment also remained more cost-effective compared to standard treatment, both in the optimistic and pessimistic scenarios.

In the optimistic scenario (25% increase in the proportion of controlled patients), the average cost per patient was lower with personalized treatment compared to standard care (16 887€ vs 51 890€), representing a saving of 35 003€ per patient. The proportion of controlled patients was 13% higher with personalized treatment (91% vs 78%). The ICER indicated that for each additional patient controlled with personalized treatment, a saving of 261 718€ was achieved compared to standard treatment per patient in a scenario of 2 years of treatment. In the worst-case scenario (25% reduction in the proportion of controlled patients), the average cost per patient was lower with personalized treatment compared to standard therapy (16 324€ vs 55 803€), representing a saving of 36 479€ per patient. The proportion of controlled patients was 18% higher with personalized treatment (87% vs 69%). The ICER indicated that for each additional patient controlled with personalized treatment, a saving of 219 577€ was achieved compared to standard treatment in a 2-year estimation.

## Discussion

These results from the ACROFAST study demonstrate that personalized medical treatment in acromegaly is not only clinically effective but also cost-efficient compared to conventional treatment approaches. Our findings highlighted that the personalized treatment strategy, which guides therapeutic decisions based on predictive biomarkers, achieves normal IGF1 control more frequently and in a significantly shorter timeframe than the conventional trial-and-error approach. This approach was emphasized some years ago by Melmed supporting an integrative medicine–based clinical practice in which this acceleration of the disease control translated into reduced healthcare costs and improved patient outcomes [18].

The cost-effectiveness of personalized treatment in acromegaly stems from its ability to optimize drug selection and dosing from the outset with relatively low costs (121€ per patient) in comparison with other diseases that require genetic studies [19]. In this context, for an affordable cost, an individualized approach has the ability to minimize the need for iterative adjustments that are common in the assay-error methodology [4]. By achieving higher and faster biochemical normalization, patients spend less time under suboptimal disease control, which reduces the risks associated with prolonged exposure to elevated GH and IGF1 levels, such as cardiovascular complications and metabolic dysregulation [20]. The costs related to the biomarkers analysis as well as the differences among both groups would have been different if the study would have been performed in other country, as the costs applied in the Spanish national health services are substantially different compared to other European countries. In this context, in other countries with higher unitary costs, the differences would have been much higher. Thus, in a certain way, the ACROFAST study was designed under less favorable conditions for comparing costs with the standard treatment, which gives even more value to the results obtained.

Importantly, the cost savings observed in this study are independent of the clinical superiority of personalized treatment in achieving biochemical control. Even if the clinical outcomes would have been equivalent between personalized and standard approaches, the reduced time to normalization inherently decreases costs associated with additional consultations, laboratory tests, and adjustments in therapy. Furthermore, personalized medicine fosters patient-centered care, potentially improving adherence and satisfaction, which are critical for chronic disease management and reduces the risk of treatment failure [21, 22]. To sum up, the personalized approach promotes patient empowerment and engagement in their care. This empowerment is also valid for physicians, as the understanding of the disease at the individual level that the study of biomarkers provides goes against therapeutic inertia and encourages medical practitioners to treat the patient with an appropriate medication. From a healthcare systems perspective, the efficient allocation of resources toward therapies with high predictive success enhances the sustainability of acromegaly management.

These findings underline the importance of integrating personalized approaches in the treatment of acromegaly and potentially other endocrine disorders. The combination of higher precision in drug selection and faster achievement of therapeutic goals, makes personalized medicine a way to more efficient and effective healthcare delivery [23]. Future studies should explore its applicability in broader populations and evaluate its long-term economic and clinical benefits. Moreover, implementation studies should investigate how personalized protocols perform outside clinical trials—particularly in real-world, resource-variable settings [24]. From the standpoint of healthcare systems, the more efficient allocation of resources toward treatments with higher predictive success rates improves the sustainability of managing a chronic and costly condition like acromegaly. As healthcare systems shift toward value-based care models, personalized strategies like those in ACROFAST become increasingly attractive for long-term integration [25].

The control of acromegaly and the use of pegvisomant have been related to an improvement in the metabolic risk factors related to acromegaly. A better control of the disease could have carried better control of risk factors and potentially, fewer costs related to control them. It is a limitation of the study that we cannot answer as it was not the primary objective of the ACROFAST study. However, this will not affect the results obtained in favor of personalized treatment, and in any case, the costs will be lower. As other studies have done [26, 27], we focused the cost-effectiveness analysis on the direct costs of acromegaly without including the indirect costs or the costs related to other treatments different than those used for acromegaly. Regarding the 6 patients treated with pegvisomant on monotherapy, 2 of them presented diabetes and the value of HbA1c improved at the end of the follow-up from 7.8% and 8% to 7% and 7.1%. This could be related to the control of the disease but also to an intensification of diabetes treatment or lifestyle changes.

Our study did not include the use of pasireotide in either arm, because at the time of the study's design, this compound was not yet sufficiently incorporated into the therapeutic medical strategies for acromegaly in Spain, nor had predictor biomarkers been described at the beginning of the study. This would have introduced bias into the standard treatment, so it was ultimately not included in the study protocol. However, it will be included in the ACROFAST II study, which will begin in late 2025 and it was considered apart from the study protocol in cases where hormonal control had not been achieved, as indicated in Fig. 1. Our study compared the cost-effectiveness of a personalized approach vs a standard therapy with fixed first-line drugs, thus it was not a drug cost-effectiveness study. However, some studies dealing with cost-effectiveness of specific drugs have been performed. In this regard, pasireotide has been shown as most cost-effective treatment compared with pegvisomant in case of persistent acromegaly after surgery, with a savings balance of 9423.26€/person/year (*P* = .12), that reach the amount of 30 415.98€/person/year at high doses (*P* < .001) [26]. This approach aims to use the same medication for all patients, and is surely less beneficial than individualized medical treatment. For example, using fgSRLs for responder patients, pasireotide for those with specific favorable predictive biomarkers to that compound, and pegvisomant possibly combined with radiotherapy for nonresponder patients. Also, Bonert et al [27] showed that after fgSRLs failure, low-dose fgSRLs plus weekly pegvisomant represents a good option for achieving cost-effective, optimal biochemical control in patients with uncontrolled acromegaly requiring combination therapy. Future research should build on these findings by exploring the broader application of personalized medicine in endocrine and other chronic disorders with all the drugs available for clinical practice with clearly different mechanisms of action. Comparative studies will also evaluate long-term benefits, including survival, cost-effectiveness across healthcare systems in different countries, and the broader societal impact.

In conclusion, this post hoc analysis from the ACROFAST study highlights the transformative potential of personalized medicine in the management of acromegaly, offering not only significant clinical benefits that restore more patients to a hormonally controlled state more rapidly and effectively than standard approaches but also cost savings.

## Data Availability

Some or all datasets generated during and/or analyzed during the current study are not publicly available but are available from the corresponding author on reasonable request.

## Clinical trial information

Trial Registration Number: MPD-SOM-2019-01.

## Abbreviations

fgSRL: first-generation somatostatin receptor ligand
GH: growth hormone
ICER: incremental cost-effectiveness ratio
IGF-1: insulin-like growth factor 1
MRI: magnetic resonance imaging
sAOT: short Acute Octreotide Test
WHO: World Health Organization
